# Generation of hyperlipidemic rabbit models using multiple sgRNAs targeted CRISPR/Cas9 gene editing system

**DOI:** 10.1186/s12944-019-1013-8

**Published:** 2019-03-18

**Authors:** Tingting Yuan, Yi Zhong, Yingge Wang, Ting Zhang, Rui Lu, Minya Zhou, Yaoyao Lu, Kunning Yan, Yajie Chen, Zhehui Hu, Jingyan Liang, Jianglin Fan, Yong Cheng

**Affiliations:** 1 Institute of Translational Medicine, Medical College, Yangzhou University, Yangzhou, 225001 China; 2 Affiliated Hospital of Yangzhou University, Yangzhou, 225001 China; 3 College of Veterinary Medicine, Yangzhou University, Yangzhou, 225009 China; 4 Jiangsu Key laboratory of integrated traditional Chinese and Western Medicine for prevention and treatment of Senile Diseases, Yangzhou University, Yangzhou, 225001 China; 5 Jiangsu Co-Innovation Center for Prevention and Control of Important Animal Infectious Disease and Zoonoses, Yangzhou, 225009 China; 6 Department of Molecular Pathology, Faculty of Medicine, Graduate School of Medical Sciences, University of Yamanashi, Yamanashi, 409-3898 Japan; 7 Beijing hospital, Beijing, 100730 China

**Keywords:** CRISPR/Cas9, Hypercholesterolemia, Atherosclerosis, Rabbits, Multiple sgRNAs

## Abstract

**Objective:**

To generate novel rabbit models with a large-fragment deletion of either LDL receptor (LDLR) and/or apolipoprotein (apoE) genes for the study of hyperlipidemic and atherosclerosis.

**Methods:**

CRISPR/Cas9 system directed by a multiple sgRNAs system was used in rabbit embryos to edit their LDLR and apoE genes. The LDLR and apoE genes of founder rabbits were sequenced, and their plasma lipids and lipoprotein profiles on a normal chow diet were analyzed, western blotting was also performed to evaluate the expression of apolipoprotein. Sudan IV and HE staining of aortic were performed to confirm the formation of atherosclerosis.

**Results:**

Six knockout (KO) rabbits by injection of both LDLR and apoE sgRNAs were obtained, including four LDLR KO rabbits and two LDLR/apoE double- KO rabbits. Sequence analysis of these KO rabbits revealed that they contained multiple mutations including indels, deletions, and substitutions, as well as two rabbit lines containing biallelic large fragment deletion in the LDLR region. Analysis of their plasma lipids and lipoprotein profiles of these rabbits fed on a normal chow diet revealed that all of these KO rabbits exhibited remarkable hyperlipidemia with total cholesterol levels increased by up to 10-fold over those of wild-type rabbits. Pathological examinations of two founder rabbits showed that KO rabbits developed prominent aortic and coronary atherosclerosis.

**Conclusion:**

Large fragment deletions can be achieved in rabbits using Cas9 mRNA and multiple sgRNAs. LDLR KO along with LDLR/apoE double KO rabbits should provide a novel means for translational investigations of human hyperlipidemia and atherosclerosis.

## Introduction

Hyperlipidemia is the major risk factor of atherosclerosis [1]. To study the pathogenesis of atherosclerosis and develop new therapeutics, experimental animal models are essential. Currently, several animal models have been used, including rats [2, 3], pigs [4, 5] zebrafish [6], and mices [7]. Appropriate animal models are critical not only for basic research, but also for the development of diagnostic tools. In this regard, the rabbit has become the most suitable animal model for the study of human hyperlipidemia because of its unique characteristics of lipid metabolism resembling those of humans [8]. Watanabe heritable hyperlipidemic (WHHL) rabbits develop spontaneous hypercholesterolemia and atherosclerosis due to genetic low-density lipoprotein (LDL) receptor deficiency. However, this model is difficult to obtain, which hampers the use of these rabbits for the study of atherosclerosis. Recently, apolipoprotein E (apoE) knockout (KO) rabbits [9] and low-density lipoprotein receptor (LDLR) KO rabbits [10] have been reported, but apoE and LDLR double-KO rabbits have not yet been generated. In the current study, we attempted to create double-double KO rabbits using CRISPR/Cas9 technology by aiming at large gene fragment deletions via multiple sgRNAs to edit LDLR and apoE genes. Those KO rabbits with large-fragment LDLR or LDLR/apoE gene deletions exhibited remarkable hyperlipidemia and developed aortic and coronary atherosclerosis on a normal chow diet. These results indicate that the CRISPR/Cas9 system-directed by multiple sgRNAs-can induce large fragment deletions of the LDLR gene in rabbits and that these LDLR KO and LDLR/apoE double-KO rabbits should provide novel models for elucidating the mechanisms and therapeutic interventions for hyperlipidemia and atherosclerosis.

## Materials and methods

### Animals

New Zealand White (NZW) rabbits were purchased from the Animal Genetic Engineering Laboratory at Yangzhou University. Rabbits were allowed access to food and water ad libitum. All animal studies were conducted according to the approval of the Animal Care Committee of the Yangzhou University.

### Cas9 mRNA and sgRNA preparation

**Fig. 1:**
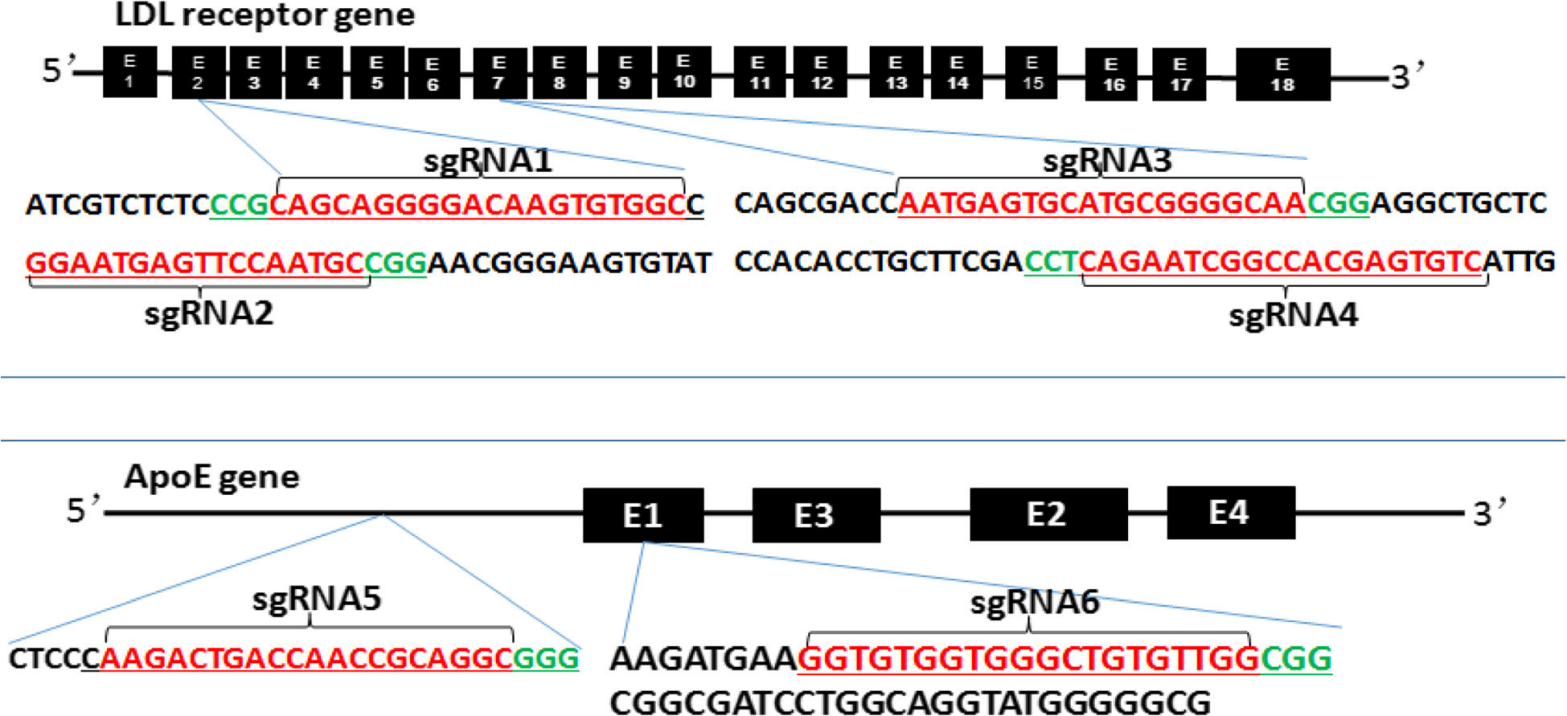
LDL receptor and apoE genes map and targeting sequences. Schematic illustration of the CRISPR/Cas9-targeting sites of rabbit LDLR and apoE genes. Exons are shown as boxes. sgRNA-targeting sequences are highlighted in red, and the protospacer-adjacent motif (PAM) sequences are in green. Four sgRNAs are designed for the LDLR gene (two sgRNAs in exon 2 and two sgRNAs in exon 7) and two sgRNAs are for the apoE gene (one sgRNA targeting the intron and one sgRNA targeting exon 1)

To design KO loci of rabbit LDLR and apoE genes, we obtained sequences from the Library of the National Center for Biotechnology Information (NCBI) (http://www.ncbi.nlm.nih.gov/) and designed the CRISPR/Cas9 sgRNAs (sgRNA) using the tool on the website of http:// crispr.mit.edu. After screening, we obtained six sgRNA targeting loci: four for the LDLR gene (designated as LDLR-sgRNA1, −sgRNA2, −sgRNA3, −sgRNA4) by anchoring to exons 2 and 7 respectively and two for apoE gene (apoE-sgRNA5 and -sgRNA6) by anchoring to intron and exon 1 as shown in Fig. 1. The protocol for CRISPR/Cas9 construction has been described in detail by our published protocols [10].

### Zygote injection and embryo transplantation

Rabbit embryos were obtained from diestrous female rabbits at an age of 6–8 months. These females were superovulated with 60 IU follicle stimulating hormone (FSH) at every 12 h (for six iterations) from 8 AM to 8 PM (60 IU in total). At the last time injection with FSH, the donors and recipients were injected with 10 IU human chorionic gonadotropin (HCG) simultaneously, and then, the donors were mated with fertile male rabbits. At 18–20 h later, embryos were flushed out using PBS and collected for microinjection. A mixed solution containing Cas9 mRNA (40 ng/ul)and multiple sgRNAs (13 ng/ul) was microinjected into the cytoplasm of embryos under a Leica inverted light microscope. The injected embryos were transferred into M2 cushion fluid and incubated at 38 °C, 5% CO_2_ for 30 min. 20–30 injected zygotes were transferred into the oviducts of pseudo-pregnant females.

### Sequencing analysis of founder rabbits

Genomic DNA was extracted from a small piece of ear biopsy using the phenol- chloroform extraction method. sgRNA target sites were amplified by PCR using the primers shown in Table 1. PCR products were purified after agarose gelel ectrophoresis and cloned into a pMDTM 19-T vector cloning kit (Takara Bio, Inc., Japan). The positive clones were sequenced and analyzed using Lasergene software (DNASTAR, Inc., U.S).

**Table 1:** Sequences of primers in this study

Name	Sequence(5′-3′)	Size	Included sgRNAs
p1	5′-GCTGTCCTCCGCTGCTTTC-3′ 5′-CAGGTCTGCTCCCACTCGTC-3’	282 bp	sgRNA1–2
p2	5′-TCAGACGAGCCCATCAAAGAG − 3′ 5′-AGGGACCCAGCCCAAACA-3’	528 bp	sgRNA3–4
p3	5′-GGGGAGACTGGAGCAGACAA-3′ 5′-GTGCGGGAGCAAAGTGGT-3′	560 bp	sgRNA5–6
p4	5′-AGGGCTGGGCTGGGAAAAAG-3′ 5′-GAGGAAGAGGCTGGGGGAGG-3’	3322 bp	sgRNA1–4

### Off-target assay

The CRISPR/Cas9 system has enabled efficient modification of gene*s* in vivo or in vitro for studying phenotypic effects of genetic perturbations. However, off-target effects are an inherent risk in this technology [11]. We screened the rabbit genome and predicted five potential off-target sites (POTS) for every sgRNA using the online CRISPR Design tool developed by Zhang and colleagues at MIT (http://crispr.mit.edu/). The primers are listed in Table 3. The PCR products of these potential off-target sites were Sanger sequenced for determining whether off-target effects occurred.

### Phenotypic examinations

#### Analysis of plasma lipids and apolipoprotein

EDTA plasma was collected from rabbits that were fasted for 16 h. Plasma total cholesterol (TC), TG, LDL-C and HDL-C were measured using enzymatic assay kits (Wako Pure Chemical Industries Ltd., Osaka, Japan). Plasma apoE, apolipoprotein B (apoB), and apolipoprotein A-I (apoA-I) were detected by Western blotting. The primary antibodies used are as follows: goat anti-apoE (Rockland, Limerick PA), sheep anti-apoA-I (AbD Serotec, Oxford, UK), and goat anti-apolipoprotein B (apoB) (Rockland, Limerick, PA) polyclonal antibodies (Abs). Immunocomplexed proteins were identified by reaction with horseradish peroxidase-conjugated donkey anti-goat IgG (Jackson Immuno Research Laboratories, West Grove, PA) and donkey anti-sheep IgG (Chemicon, Temecula, CA) polyclonal Abs.

#### Plasma lipoprotein profiles

Plasma lipoprotein profiles were analyzed using agarose gel electrophoresis. The protocol has been described in detail by our published protocols [10].

#### Analysis of atherosclerosis

We selected two rabbits among six KO founder rabbits for pathological analysis, which were 5♀ founder with LDLR/apoE double-KO and 7♂ founder with large fragment deletion. The aortic trees were isolated and opened out and, after fixing in formalin for 24 h, they were stained with Sudan IV (Wako Pure Chemical Industries Ltd., Osaka, Japan). For histological analysis, serial paraffin sections were stained with hematoxylin-eosin (HE) and immunohistochemically stained with monoclonal antibodies against either macrophages (clone: RAM11, Dako, Carpinteria, CA) or a-smooth muscle actin for smooth muscle cells (clone: HHF35, Dako, Carpinteria, CA).

## Results

### Generation of KO rabbits

**Fig. 2:**
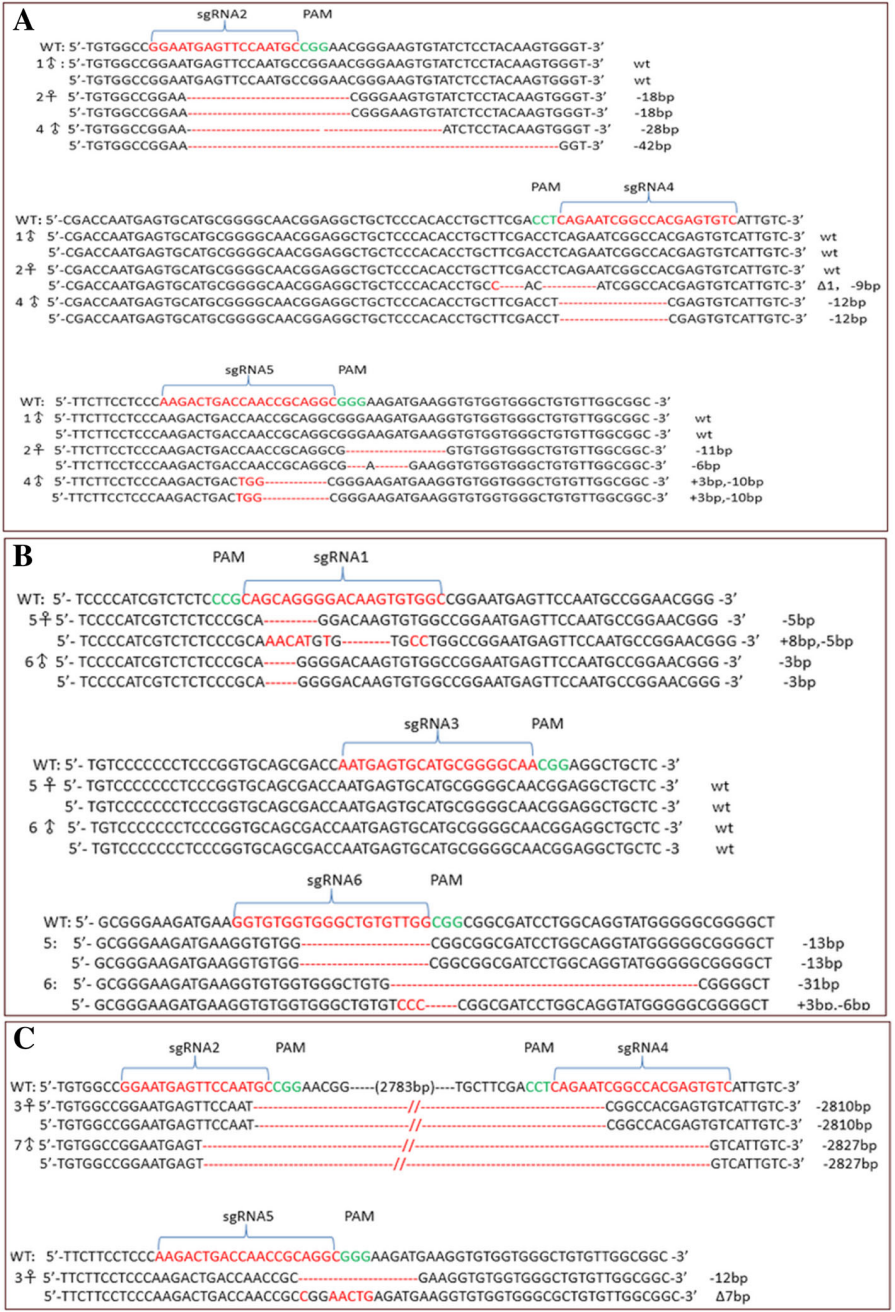
Mutations of the modified alleles were detected by sequencing. (**a**) Mutations generated by LDLR sgRNA 2, 4 and apoE sgRNA 5, primer P1, P2, P3 were used (1♂, 2♀, 4♂). (**b**) Mutations generated by LDLR sgRNA 1, 3 and apoE sgRNA 6, primer P1, P2, P3 were used (5♀, 6♂). (**c**) Mutations generated by LDLR sgRNA 2,4 and apoE sgRNA 5 (3♀) and LDLR sgRNA 1–4 (7♂), primer P4 were used. WT refers to the wild-type allele sequences, deletions (−), insertions(+)and substitutions (Δ) are shown, target sequences are red. No mutation was found in 1♂ and the sgRNA 3, 3♀ and 7♂ showed the large-fragment deletion

**Fig. 3:**
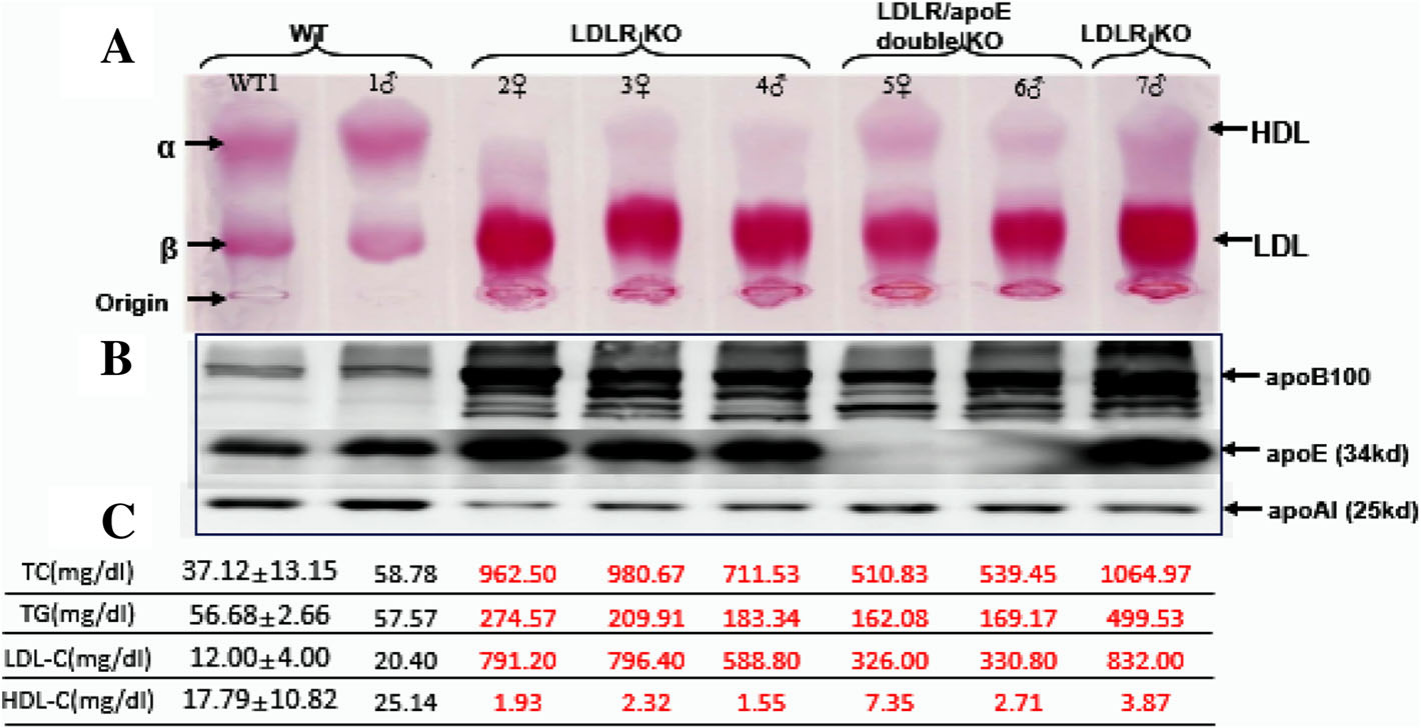
Analysis of plasma lipids and lipoproteins. (**a**) Agarose gel electrophoresis of plasma lipoproteins. 4 μl of plasma was loaded in each well and fractionated on a 1% agarose gel and stained with Fat Red 7B for neutral lipids. Lipoprotein migration positions are indicated by arrows. (**b**) Analysis of plasma apolipoproteins by Western blotting. Plasma samples (0.5 μl) were fractionated on 10% SDS-PAGE and transferred to a cellulose membrane probed with Abs against apoB, apoE and apoAI as described in the Materials and Methods section. (**c**) Plasma levels of total cholesterol (TC), triglycerides (TG), LDL-cholesterol (LDL-C) and HDL-cholesterol (HDL-C)

As shown in Fig. 1, we designed six sgRNAs: four for LDLR and two for apoE gene. In the end, we obtained seven F0 rabbits, six of which were mutated as shown in Table 2. Initially, we attempted to generate LDLR and apoE double KO rabbits by single injection of the mixtures of three sgRNAs (sgRNA 2, 4, 5) along with Cas9 mRNA. Four pups were born and three of them (2♀, 3♀, and 4♂) showed mutations in both LDLR and apoE genes, and one of them (3♀) was accompanied by a deletion of a large fragment in the LDLR gene shown in Fig. 2. Although all of them showed hyperlipidemia, apoE was still detected in the plasma by Western blotting (Fig. 3). Therefore, apoE mutations generated by sgRNA 5 may not be sufficient to inhibit apoE expression. The 1♂ was male and did not show any genetic mutations, and its plasma levels of lipids were completely normal compared to that of normal wild-type (WT) rabbits, as shown in Table 4. For the second experiment, we injected mixtures of three sgRNAs (sgRNA 1, 3, 6) along with Cas9 mRNA and generated two founders (5♀ and 6♂). Although no mutation was detected on the sgRNA 3-targeted locus of either founder, these founders exhibited hyperlipidemia and no apoE was detected in the plasma (Fig. 3b), suggesting that both founders were double KOs. Finally, we tried to generate an LDLR single KO by injection of four LDLR sgRNAs (sgRNA 1, 2,3, 4). Eventually, we obtained one founder (7♂) with the large deletion of the gene, also similar to that founder 3♀. Compared with other founders, 3♀ and 7♂ rabbits showed marked hypercholesterolemia (Fig. 3, Table 4).

**Table 2:** Summary of gene targeting efficiency

Microinjected sgRNAs	sgRNA 2,4,5	sgRNA 1,3,6	sgRNA 1,2,3,4	Total
No. of embryos collected	113	137	179	429
No. of embryos injected	96	109	140	345
No. of recipients	5	5	6	16
No. of gestations	3	3	4	10
No. of pups born	10	3	5	18
No. of live pups	4	2	1	7
No. of mutant pups	3	2	1	6
No. of pups with large fragment deletion	1	0	1	2
Rate of pregnancy	60.0%(3/5)	60.0%(3/5)	66.7%(4/6)	62.5%(10/16)
Rate of mutations	75.0%(3/4)	100.0%(2/2)	100.0%(1/1)	85.7%(6/7)

**Table 3:**
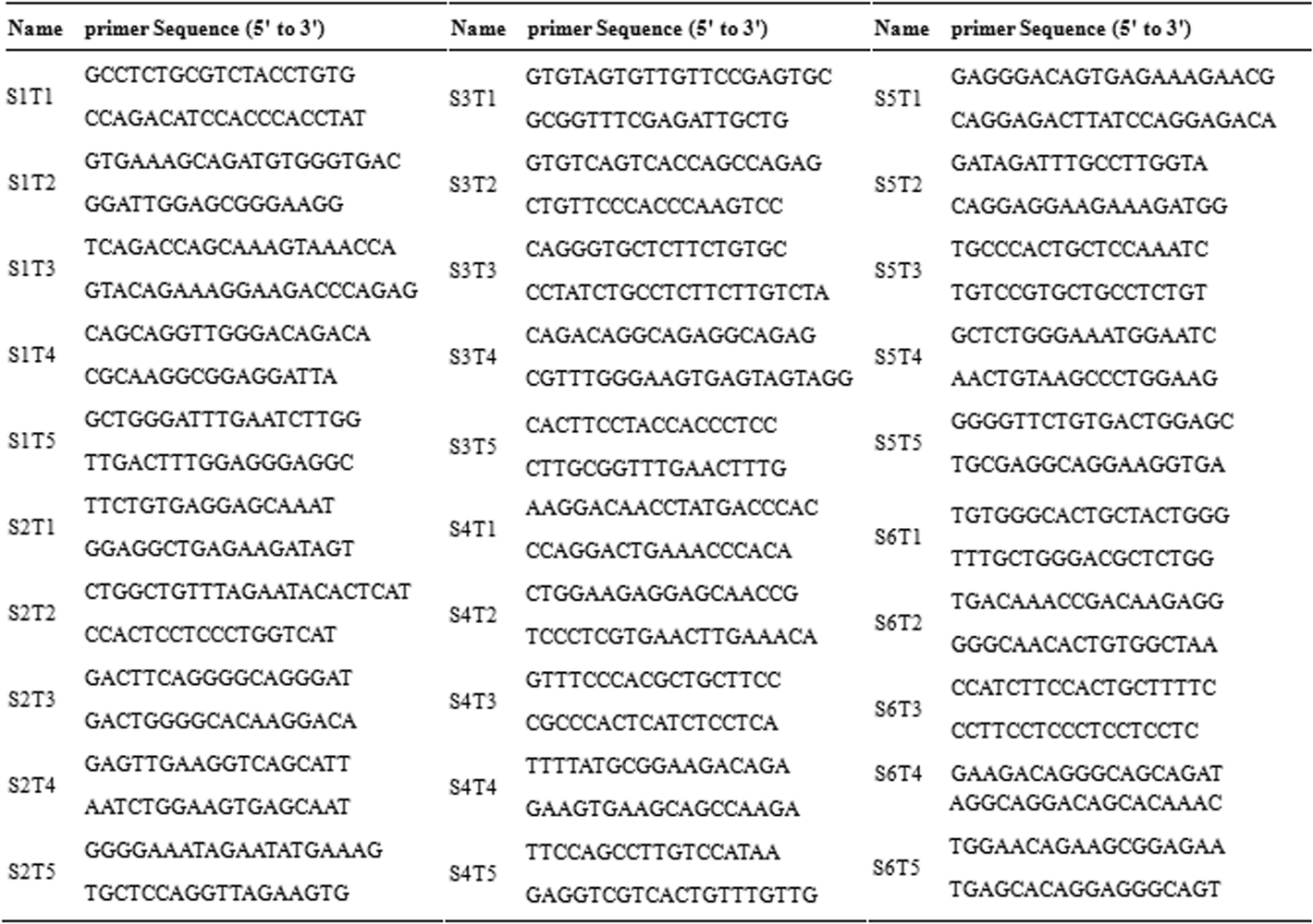
The primers of potential off-target sites

### Off-target validation

To verify whether multiple sgRNAs can cause any off-targeting effects, we examined 30 POTS in total, Primers are listed in Table 3. We sequenced and analyzed the potential off-target sites of these mutant rabbits. The results showed that there were no mutations, indicating that they did not have off-target effects within our detection range.

### Analysis of plasma lipids and lipoproteins

On a normal chow diet, six founder KO rabbits at the age of 12 weeks exhibited hyperlipidemia (except 1♂) and their plasma TC, TG, LDL-C levels were significantly higher than those of WT rabbits (Fig. 3c). Among them, 3♀ and 7♂ -which had large fragment deletions-exhibited the most serious hyperlipidemia: TC levels were increased by 26-fold and the LDL-C levels were increased by 52-fold compared with those in WT rabbits. Although LDLR/apoE double-KO 5♀ and 6♂ exhibited relatively moderate hyperlipidemia, their TC and LDL-C levels were also elevated 14 times and 20 times greater than those of WT rabbits. In addition to the above, the plasma lipid levels of LDLR KO founders were also much more severe than those of LDLR/apoE double-KO founders, which is most likely related to their type of gene mutation that the mutation type of LDLR gene in double-KO rabbits is relatively simple and the role of LDLR is more essential than apoE in lipid metabolism regulation. Because all these six founder rabbits exhibited hyperlipidemia, we further analyzed plasma lipoproteins profiles. Agarose gel electrophoresis revealed that increased plasma lipids in founder rabbits on a chow diet were essentially caused by increased lipoproteins migrated from the original position to the β-area, assuming that they were those of chylomicron (very low-density lipoprotein, VLDL), and especially LDL particles (Fig. 3a). Simultaneously, western blotting results showed that plasma apoB (apoB 100 and apoB 48 included) and apoE (except the 5♀ and 6♂ with apoE proteins were deleted because of the injection of sgRNA6) increased while the apoAI decreased in founder rabbits (Fig. 3b), which verified the previous analysis on the formation of hyperlipidemia.

### Assessment of atherosclerosis

**Fig. 4:**
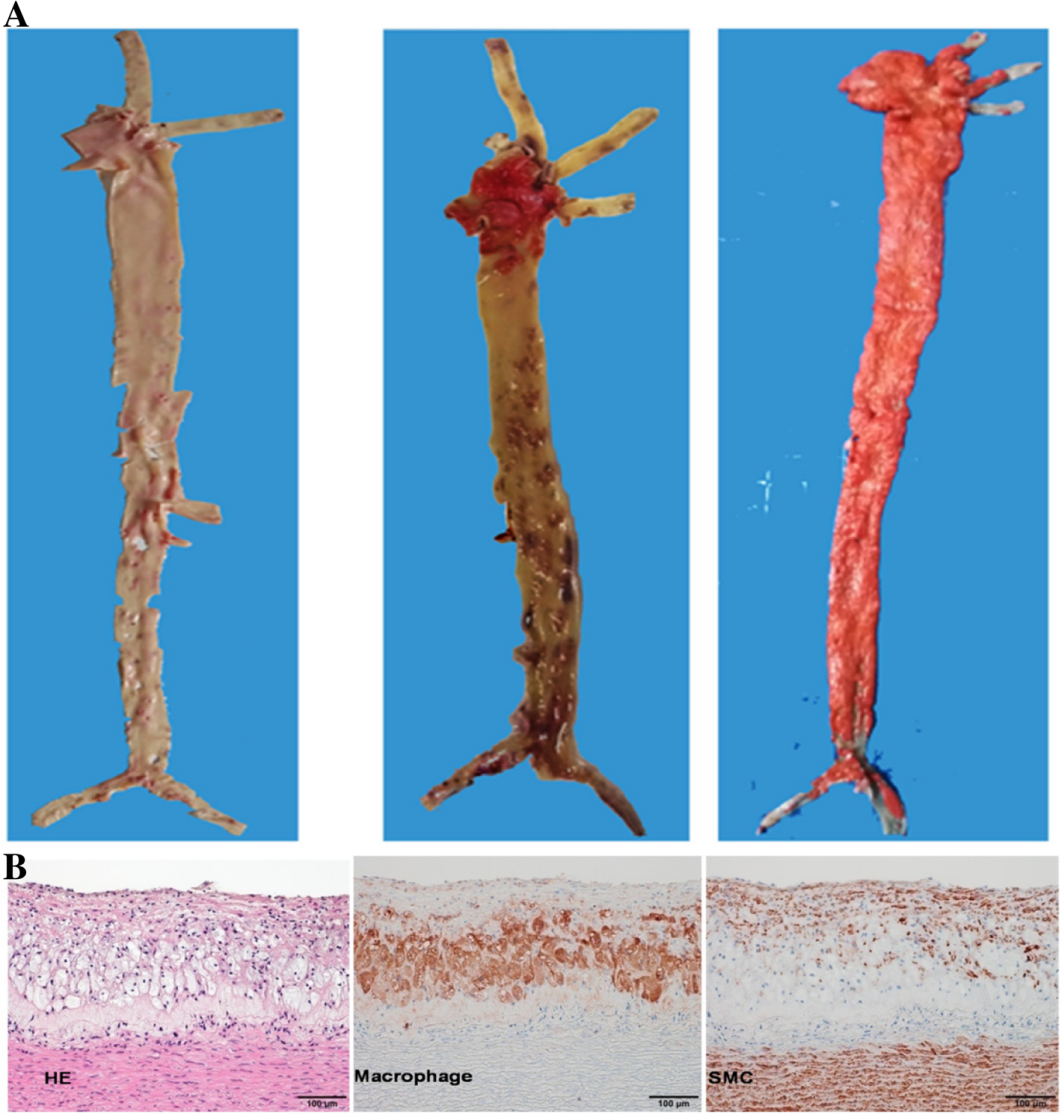
Pathological analysis of aortic atherosclerosis in KO rabbits. (**a**) Gross lesions of aortic atherosclerosis of KO rabbits (5♀, 7♂) stained with Sudan IV. The normal aorta is cut open and stained by Sudan IV to show as a reference (left) to the other two (5♀ and 7♂, respectively). Aortas stained by Sudan IV show different degrees of aortic lesions (red areas stained with Sudan IV). (**b**) Micrographs of aortic atherosclerosis. Serial sections of paraffin specimens are stained with hematoxylin and eosin (HE) or immunohistochemically stained with Abs against either RAM-11 (macrophages) or smooth muscle β-action (smooth muscle cells, SMC). The lesions are composed of macrophage-derived foam cells intermingled with smooth muscle cells

Elevated levels of lipoproteins can drive the development of atherosclerosis in humans and animals. We obtained six KO rabbits including four LDLR KO (two of them with allelic large fragment deletions) rabbits and two LDLR/apoE double KO rabbits. Because our previous article [10] introduced the arterial lesions of LDLR KO rabbits, currently, we focused on the 7♂ — with a large fragment deletion and accompanying severe hyperlipidemia —and the 5♀ rabbit with LDLR/apoE double- KO. We opened up their aortas, following by Sudan IV staining, and examined their histological analysis with HE staining. As shown below (Fig. 4a), aortic atherosclerotic lesions were assembled and mainly seen on the aortic arch. Microscopically, the accumulated atherosclerotic lesions consisted almost completely foam cells derived macrophage in the center and smooth muscle cells on the top, and the 7♂ rabbit apparently had more lesions than the 5♀ rabbit which almost covered the entire arterial tree (Fig. 4b).

## Discussion

Hyperlipidemia is a common genetic disorder inherit by genetic mutations mostly in LDLR, apoB-100, or proprotein convertase subtilisin kexin type 9 (PCSK9) loci which can result in xanthoma, atherosclerosis, coronary atherosclerotic heart disease (CAD), and other cardiovascular diseases [12]. The majority (60–80%) of the patients with family hyperlipidemia (FH) harbor mutations in the *LDLR* gene [13], but apoE serves as a major member of many ligands of LDLR which can transport plasma chylomicrons and VLDL cholesterol to the liver for metabolism by LDL receptor and related receptors. As a result, LDLR and apoE, especially the LDLR, play a critical role for the clearance of lipoprotein remnant. From the cholesterol-fed rabbits [14] to the appearance of the Watanabe heritable hyperlipidemic (WHHL) rabbits established in 1980 by Watanabe Y [15], the animal models like the genetically edited hyperlipidemia model have also emerged with the development of novel gene editing tools. In the our most recently published study, we reported an LDLR KO rabbit model —but with no large fragment deletion —which showed severe spontaneous hyperlipidemia and atherosclerosis [10]. Whereas, Manabu Niimi and colleagues [9] showed that homozygous (but not heterozygous) apoE KO rabbits showed mild hyperlipidemia, but when challenged with a cholesterol diet, exhibited greater diet-induced hyperlipidemia. In our present study, we established LDLR/apoE double-KO rabbit and showed that the hyperlipidemia in LDLR/apoE double KO founders are milder than those of LDLR KO founders. This result most likely related to their mutation type of the LDLR gene and implies that the role of LDLR is more essential than that of apoE in lipid metabolism regulation.

The CRISPR/Cas9 system was developed to edit the genome, and even the utility of it for gene therapy in humans has been recognized and extensively investigated [16]. With the off-target damage now being limited by the development of sensitive detection technology and improved methods [17–19], the CRISPR/Cas9 system has become a routine procedure for functional analysis of genetic pathways, both in cellular culture and in animal models. However, most mutations reported are small insertions and deletions or point mutations. Now, it is not uncommon to edit multiple genes directed by multiple sgRNAs at the same time, which may also cause large fragments deletion of genes [20–22]. Recently some findings have also demonstrated that it is effective for the deletion of large genomic segments in many organisms and cell types, including rice [23], *Saccharomyces cerevisiae* [24], *Escherichia coli* [25], mouse, rat [26] and human [27]. In 2016, Y. Song and colleagues [28] first described a large biallelic gene deletion in rabbit by the dual sgRNAs system and accompanied it with a typical albino phenotype. Also Zheng, Q.P. and colleagues [29] showed that this system can efficiently create DNA deletions of up to 10 kb by transient transfection with two sgRNAs plasmids coupled with Cas9 plasmid in HEK 293 T cells, and that the repair of this deletion process is largely accomplished by precise end joining. Based on these, to disable LDLR function induced by the large fragment deletion we microinjected multiple sgRNAs anchored between exon 2 encoded the ligand binding domain and exon 7 encoded the homologous epidermal growth factor precursor homology of rabbit LDLR gene. Our results imply that it is possible to achieve large fragment (2810 kb and 2827 kb, including five exons) deletion from chromosomes of rabbits. Undoubtedly, the hyperlipidemia phenotype that occurs from large fragment deletion is much higher than in other models, which indicates that this approach can provide a new option for the gene therapy for intractable diseases.

LDLR, a transmembrane cellular protein, plays a crucial role in the receptor-mediated pathway of lipoprotein metabolism [30]. The human *LDLR* gene consists of 18 exons and 17 introns with a length of approximately 45 kb [31]. These exons encode five functional domains, the most important and relevant being the ligand binding domain and the epidermal growth factor precursor-homology domain (EGF homology domain). The former domain of the mature receptor contains the binding site for apoB, apoE, and related lipoproteins [32]. It can transport the lipoprotein from the cell surface to the lysosome to be hydrolyzed, and then the serum cholesterol levels can be controlled to a certain extent. When this domain loses its function or dysfunction, it will affect or even block the entry of plasma lipids into the hepatocytes for metabolism, which naturally causes elevated blood lipids. The first epidermal growth factor-like repeat (EGF-A) in the EGF homology domain of LDLR can bind with proprotein convertase subtilisin/kexin type 9 (PCSK9) which promotes degradation of hepatic LDLR. As a consequence, the LDLR is rerouted from the endosome to the lysosome, where it is degraded and results in redistribution of the receptor from the plasma membrane to lysosomes [33]. Therefore, this domain regulates blood lipids by affecting the redistribution of LDLR on the surface of hepatocytes, and now it is also the hot therapeutic target for hyperlipidemia treatment: the new drug pcsk-9 inhibitor can lower lipid [34]. Based on this, in our research, we may successfully reduced the quantity of LDLR on the surface of hepatocytes and disable the LDLR, and then establish the hyperlipidemia model. But compared to WHHL rabbits, our founders show robust differences: their LDLR gene mutations are located in the exon 2 and exon 7 and the types of mutations are diverse and, more importantly, it is accompanied by apoE mutations; the WHHL rabbits comprise a deletion in exon 4 of the LDLR gene that encodes a 4-amino-acid deletion in the cysteine-rich ligand-binding domain [32]; their spontaneous hyperlipidemia developed under a normal diet is far more serious than with a high cholesterol diet induced WHHL [35, 36].

Although we have successfully generated LDLR and LDLR/apoE double KO rabbit model with the spontaneous hyperlipidemia, but their genetic mutations are different from patients and also their plasma lipids are significantly higher than patients, which may limit their subsequent clinical translational applications. And for providing a reliable basis for hyperlipidemia therapy, the further research on pathogenesis of our models should be performed. In addition, the off-target effect in CRISPR/Cas9 system is a critical concern. However, this side effect was not found in our study that we supposed the Web-based algorithms may miss the potential off-target sites with less sequence similarity or such effect didn’t appear. In all, our animal models with its typical atherosclerosis and spontaneous hyperlipidemia provide novel models for the study of familial hyperlipidemia and cardiovascular disease.

## Conclusions

We generate the LDLR/apoE double KO rabbits and achieved an ~ 3 kb large gene fragment deleted from LDLR gene in the rabbit model. The LDLR KO and LDLR/apoE double-KO rabbits will be useful for further studies investigating spontaneous hyperlipidemia and related diseases.

## Abbreviations

apoB-100: Apolipoprotein B-100
APOE: Apolipoprotein E
FH: Family hyperlipidemia
HDL-C: High density lipoprotein cholesterol
KO: Knockout
LDL-C: Low density lipoprotein cholesterol
LDLR: Low density lipoprotein receptor
PCSK9: Proprotein convertase subtilisin kexin type 9
TC: Total cholesterol
TG: Triglycerides
WHHL: Watanabe heritable hyperlipidemic
